# Barriers to timely diagnosis of interstitial lung disease in the real world: the INTENSITY survey

**DOI:** 10.1186/s12890-017-0560-x

**Published:** 2018-01-17

**Authors:** Gregory P. Cosgrove, Pauline Bianchi, Sherry Danese, David J. Lederer

**Affiliations:** 1 National Jewish Health, 1400 Jackson Street, Denver, CO 80206 USA; 2 Pulmonary Fibrosis Foundation, 230 E. Ohio Street, Suite 500, Chicago, IL 60611 USA; 3 Veracyte, Inc., 6000 Shoreline Court, Suite 300, South San Francisco, CA 94080 USA; 4 Outcomes Insights, Inc., 2801 Townsgate Road, Suite 30, Westlake Village, CA 91331 USA; 5 Departments of Medicine and Epidemiology, Columbia University Medical Center, 622 W 168th S, PH14-101, New York, NY 10032 USA

**Keywords:** Interstitial lung disease, Idiopathic pulmonary fibrosis, Diagnosis

## Abstract

**Background:**

The diagnosis of idiopathic pulmonary fibrosis (IPF) and other interstitial lung diseases (ILD) presents significant clinical challenges. To gain insights regarding the diagnostic experience of patients with ILD and to identify potential barriers to a timely and accurate diagnosis, we developed an online questionnaire and conducted a national survey of adults with a self-reported diagnosis of ILD.

**Methods:**

A pre-specified total of 600 subjects were recruited to participate in a 40-question online survey. E-mail invitations containing a link to the survey were sent to 16 427 registered members of the Pulmonary Fibrosis Foundation. Additionally, an open invitation was posted on an online forum for patients and caregivers (www.inspire.com). The recruitment and screening period was closed once the pre-defined target number of respondents was reached. Eligible participants were adult U.S. residents with a diagnosis of IPF or a non-IPF ILD.

**Results:**

A total of 600 eligible respondents met the eligibility criteria and completed the survey. Of these, 55% reported ≥ 1 misdiagnosis and 38% reported ≥ 2 misdiagnoses prior to the current diagnosis. The most common misdiagnoses were asthma (13.5%), pneumonia (13.0%), and bronchitis (12.3%). The median time from symptom onset to current diagnosis was 7 months (range, 0–252 months), with 43% of respondents reporting a delay of ≥ 1 year and 19% reporting a delay of ≥ 3 years. Sixty-one percent of respondents underwent at least one invasive diagnostic procedure.

**Conclusions:**

While a minority of patients with ILD will experience an appropriate and expedient diagnosis, the more typical diagnostic experience for individuals with ILD is characterized by considerable delays, frequent misdiagnosis, exposure to costly and invasive diagnostic procedures, and substantial use of healthcare resources. These findings suggest a need for physician education, development of clinical practice recommendations, and improved diagnostic tools aimed at improving diagnostic accuracy in patients with ILD.

**Electronic supplementary material:**

The online version of this article (10.1186/s12890-017-0560-x) contains supplementary material, which is available to authorized users.

## Background

Interstitial lung disease (ILD) encompasses a broad and diverse group of diffuse parenchymal lung disorders that are characterized by alveolar inflammation, fibrosis, and other cellular changes. Of the more than 150 recognized ILDs, idiopathic pulmonary fibrosis (IPF) is among the most common and the most lethal [1, 2]. The clinical course is characterized by progressive exertional dyspnea and a decline in pulmonary function that severely limits routine physical activity [3]. Two recently approved therapies have been shown to attenuate the decline in pulmonary function [4, 5]; however, neither agent is a cure and neither agent improves lung function, symptoms, or quality of life. Timely and accurate diagnosis could potentially improve outcomes by permitting earlier initiation of antifibrotic therapy, avoiding exposure to harmful or ineffective treatments, and improving access to important treatments that improve quality of life, such as supplemental oxygen and pulmonary rehabilitation [6].

The diagnosis of IPF is challenging for even the most experienced clinicians. Accurate diagnosis requires careful exclusion of alternative etiologies and skillful integration of findings from clinical, radiologic, and pathologic exams [3, 7]. Current diagnostic guidelines define characteristic radiologic and histopathologic features that suggest a diagnosis of IPF [3]; however, high resolution computed tomography (HRCT) scans and lung biopsies frequently exhibit mixed or discordant patterns, and findings in patients with IPF and other ILDs are often marked by subtle differences [7–9]. Studies evaluating diagnostic agreement among pulmonologists, radiologists, and pathologists have reported only modest inter-observer agreement, even among expert observers [7, 10, 11]. Additionally, early diagnosis is further complicated by the insidious onset and nonspecific nature of the initial symptoms, which patients often initially attribute to age or deconditioning, leading to a delay in seeking medical attention [12].

To gain insights regarding the diagnostic experience of patients with ILD and identify potential barriers to a timely and accurate diagnosis, we developed a structured online questionnaire and conducted a national survey of adults with a self-reported diagnosis of ILD.

## Methods

A structured online survey (The INTENSITY Survey) was developed to collect data on the diagnostic experiences of adults with ILD. The quantitative survey comprised 40 defined-choice and open-ended questions related to the following topics: demographics, current and prior diagnoses, initial onset and duration of symptoms, the number and type of physicians consulted, diagnostic tests and procedures, medication history, comorbidities, and quality of life. For the latter, respondents were asked to indicate the degree to which they agreed or disagreed with a series of statements designed to elicit information on the following domains: emotional, physical, financial, personal, and professional (the full survey is available in Additional file 1).

E-mail invitations containing a link to the online survey were sent to 16 427 registered members of the Pulmonary Fibrosis Foundation, a nonprofit advocacy, education, and research organization dedicated to serving people living with pulmonary fibrosis, their caregivers, and healthcare professionals. Additionally, an open invitation was posted on the website of Inspire™ (www.inspire.com), an online health community and support network designed to connect patients and caregivers with common interests. Survey participants were offered the choice of a nominal honorarium in the form of a $20 gift card or a donation in the equivalent amount to the Pulmonary Fibrosis Foundation. The recruitment and screening period closed once the pre-defined target of 600 eligible respondents completed the survey.

Eligible participants were U.S. residents with a diagnosis of IPF or a non-IPF ILD (a full list of ILD diagnoses is available in Additional file 1). At the start of their online participation, prospective participants were informed about the content and purpose of the survey and advised that their responses would be anonymous. Screening for eligibility was accomplished by means of three online screening questions which prospective participants were required to complete prior to gaining access to the research questions.

The survey was conducted in full compliance with the American Association for Public Opinion Research best practices for research, including the reporting of outcome measures [13]. Completed survey results were tabulated by an independent research organization (Outcomes Insights Inc., Thousand Oaks, CA). Data are summarized descriptively and presented as the median (range) for continuous variables and number and percentage of respondents for categorical variables.

## Results

### Survey response

**Fig. 1:**
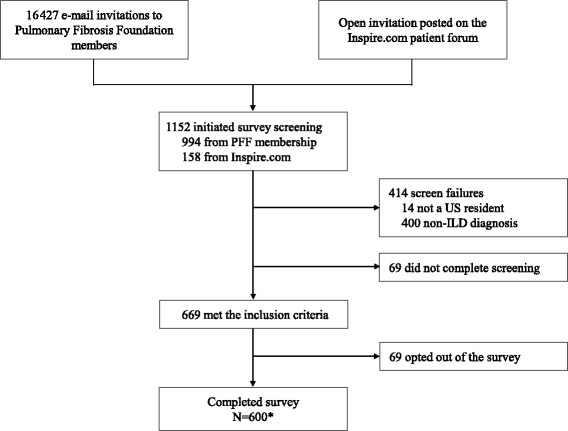
Survey response summary. ^*^Includes 503 respondents from the Pulmonary Fibrosis Foundation membership and 97 respondents from the open invitation posted on the Inspire.com patient forum

The survey response metrics are summarized in Fig. 1. Between August 14 and August 26, 2015, a total of 1152 individuals entered the online survey and initiated screening. Of these, 414 did not meet eligibility criteria, 69 did not complete screening, and 69 chose not to participate, resulting in a total of 600 respondents from 47 states who completed the survey. Among those who completed the survey, 503 (83.8%) were registered members of the Pulmonary Fibrosis Foundation and 97 (16.2%) were recruited through the open invitation on the Inspire™ online patient forum.

### Demographics and respondent characteristics

Demographics and respondent characteristics are summarized in Table 1. There was an equal distribution of male and female respondents; the median age was 69 years (range, 16–91) in males and 62.5 years (21–89) in females. IPF was the most common diagnosis (46.5%), followed by ILD (32.5%), non-specific interstitial pneumonia (15.5%), hypersensitivity pneumonitis (4.5%), and sarcoidosis (1.0%). More than two-thirds (70.1%) of respondents were diagnosed within the last 5 years. The most commonly reported comorbid conditions were gastroesophageal reflux disease (43.3%), sleep apnea (22.3%), allergy (20.7%), and cardiovascular disease (20.3%).

**Table 1:** Respondent characteristics

Characteristic	Total (*N* = 600)	IPF (*N* = 279)	Non-IPF (*N* = 321)
Median age (range), years			
Male	69 (16–92)	70 (29–92)	66 (16–83)
Female	62 (21–94)	64 (21–84)	61 (30–94)
Gender, n (%)			
Male	300 (50.0)	164 (58.8)	136 (42.4)
Female	300 (50.0)	115 (41.2)	185 (57.6)
Diagnosis, n (%)			
Idiopathic pulmonary fibrosis	279 (46.5)	279 (100.0)	0 (0.0)
Interstitial lung disease ^*^	195 (32.5)	0 (0.0)	195 (60.7)
Non-specific interstitial pneumonia	93 (15.5)	0 (0.0)	93 (29.0)
Hypersensitivity pneumonitis	27 (4.5)	0 (0.0)	27 (8.4)
Sarcoidosis	6 (1.0)	0 (0.0)	6 (1.9)
Time since diagnosis, n (%)			
≤ 6 months	32 (5.3)	18 (6.5)	14 (4.4)
> 6 months to 1 year	59 (9.8)	33 (11.8)	26 (8.1)
> 1 to 2 years	126 (21.0)	61 (21.9)	65 (20.2)
> 2 to 5 years	204 (34.0)	93 (33.3)	111 (34.6)
> 5 to 10 years	127 (21.2)	57 (20.4)	70 (21.8)
> 10 years	52 (8.6)	17 (6.1)	35 (10.9)
Comorbid conditions, n (%)			
Gastroesophageal reflux disease	260 (43.3)	115 (41.2)	145 (45.2)
Sleep apnea	134 (22.3)	68 (24.4)	66 (20.6)
Allergy	124 (20.7)	52 (18.6)	72 (22.4)
Cardiovascular disease	122 (20.3)	59 (21.1)	63 (19.6)
Pulmonary hypertension	103 (17.2)	37 (13.3)	66 (20.6)
Other pulmonary conditions ^†^	356 (59.3)	156 (55.9)	200 (62.3)
Family history of ILD, n (%)			
Yes	102 (17.0)	54 (19.4)	48 (15.0)
No	498 (83.0)	225 (80.6)	273 (85.0)

^*^Includes interstitial lung disease due to an autoimmune or connective tissue disease, respiratory bronchiolitis-interstitial lung disease, desquamative interstitial pneumonia, cryptogenic organizing pneumonia, lymphoid interstitial pneumonia, idiopathic pleuroparenchymal fibroelastosis, interstitial lung disease due to occupational exposure, radiation therapy-induced interstitial lung disease, and drug-related interstitial lung disease

^†^ Includes chronic obstructive pulmonary disease, asthma, chronic bronchitis/bronchiolitis, bronchiectasis, emphysema, pneumonia, cystic fibrosis, pulmonary edema, lung cancer, and tuberculosis

*ILD* = interstitial lung disease; *IPF* = idiopathic pulmonary fibrosis

### Diagnostic experience

Survey results indicated that most respondents experienced a gradual onset of symptoms, which typically included shortness of breath (77.2%), cough (53.0%), and fatigue (38.0%) (Table 2). The median reported time from initial onset of symptoms to the first doctor visit was 3 months, with 72% of respondents indicating that they initially attributed some or all of their respiratory symptoms to advancing age. Nearly all survey participants initially consulted a primary care physician; of these, 27.8% were referred to a specialist after the first visit, but 30.4% reported ≥ 4 visits to a primary care physician prior to being referred to a specialist (Table 2).

**Table 2:** Initial presentation

	Respondents (*N* = 600)
Initial symptoms, n (%)	
Shortness of breath	463 (77.2)
Cough	318 (53.0)
Fatigue/weakness	228 (38.0)
Chest discomfort	104 (17.3)
Unexplained weight loss	40 (6.7)
Loss of appetite	33 (5.5)
Pneumonia	13 (2.2)
Other	58 (9.7)
Time between initial symptoms and first doctor visit, n (%)	
0–5 months	356 (59.3)
6–11 months	101 (16.8)
12–23 months	64 (10.7)
24–35 months	42 (7.0)
36–47 months	14 (2.3)
≥ 48 months	23 (3.8)
Initial medical consultation, n (%)	
Primary care physician	533 (88.8)
Specialist	67 (11.2)
Primary care visits prior to referral, n (%)^*^	
1	148 (27.8)
2	99 (18.6)
3	99 (18.6)
4	35 (6.6)
> 4	127 (23.8)
Do not recall	23 (4.3)
No specialist referral	2 (0.4)

^*^ *N* = 533

**Fig. 2:**
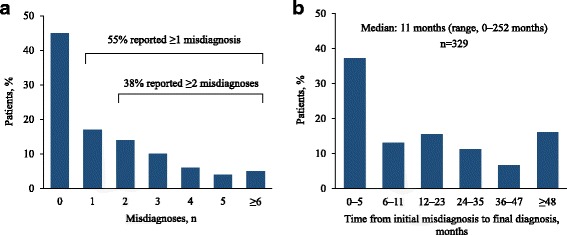
Reported frequency of misdiagnosis (**a**) and time from initial misdiagnosis to final diagnosis (**b**)

**Fig. 3:**
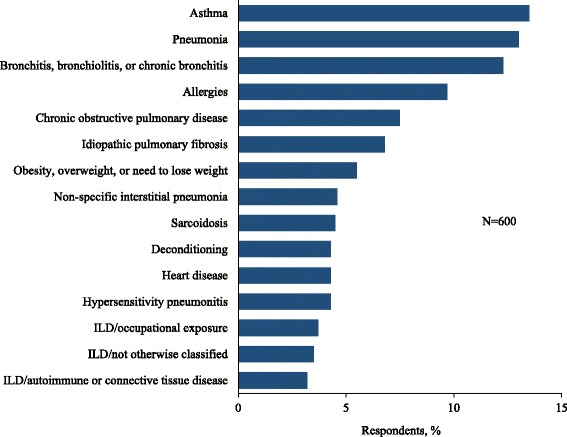
Most commonly reported misdiagnoses (reported by > 3% of respondents). *ILD* = interstitial lung disease

More than half of all respondents (55%) reported at least one misdiagnosis and more than one-third (38%) reported ≥ 2 misdiagnoses prior to the current diagnosis (Fig. 2a). Among those who were initially misdiagnosed, the median time between the initial misdiagnosis and the final diagnosis was 11 months, with 34% reporting a delay of ≥ 2 years (Fig. 2b). The most common misdiagnoses were asthma (13.5%), pneumonia (13.0%), and bronchitis (12.3%) (Fig. 3).

**Fig. 4:**
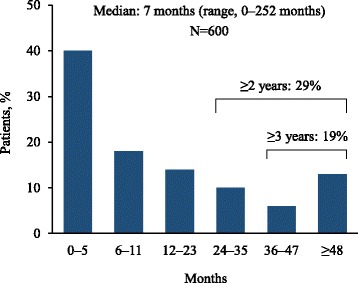
Time from initial onset of symptoms to final diagnosis

The median time from initial onset of symptoms to final diagnosis was 7 months (range, 0–252). Forty-three percent of respondents reported a delay of ≥ 1 year between the initial onset of symptoms and the final diagnosis, and nearly one in five reported experiencing a delay of ≥ 3 years (Fig. 4). Among those who underwent an invasive diagnostic procedure, the median time from symptom onset to final diagnosis was 9 months (range, 0–252).

**Fig. 5:**
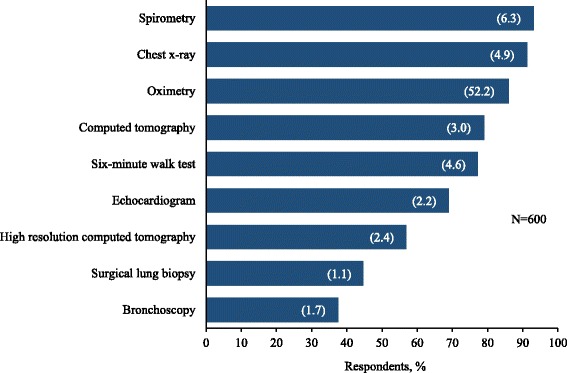
Diagnostic investigations.  Data are presented as percentage of respondents, with the mean frequency per respondent in parentheses. The mean frequency is based on the number of respondents reporting the corresponding diagnostic investigation

The majority of respondents (75%) consulted at least three physicians prior to receiving their current diagnosis. Commonly reported diagnostic tests and procedures included spirometry, chest x-ray, oximetry, computed tomography (CT)/HRCT, and the six-minute walk test (Fig. 5). Imaging studies, exercise tests, and spirometry were commonly repeated multiple times prior to the final diagnosis. Additionally, 61% reported undergoing an invasive diagnostic procedure, including 43.3% who had a surgical lung biopsy and 35.7% who had at least one bronchoscopy.

A substantial majority of respondents (88%) indicated that their final diagnosis was made by a pulmonologist, with 34.7% reporting that the diagnosis was made by a physician at a nationally recognized ILD center. Consulting a specialist with expertise in interstitial lung disease was cited by 68% of respondents as the most important contributing factor in obtaining a clear diagnosis.

Systemic corticosteroids were prescribed to 21.8% of survey participants during the diagnostic evaluation. Among those with a self-reported diagnosis of IPF, 21.1% reported prior treatment with systemic corticosteroids. Other commonly prescribed medications during the interval between initial medical consultation and the current diagnosis were antibiotics (32.0%), salmeterol/fluticasone (17.8%), N-acetylcysteine (13.5%), omeprazole (11.8%), and albuterol (11.6%).

### Quality of life

**Fig. 6:**
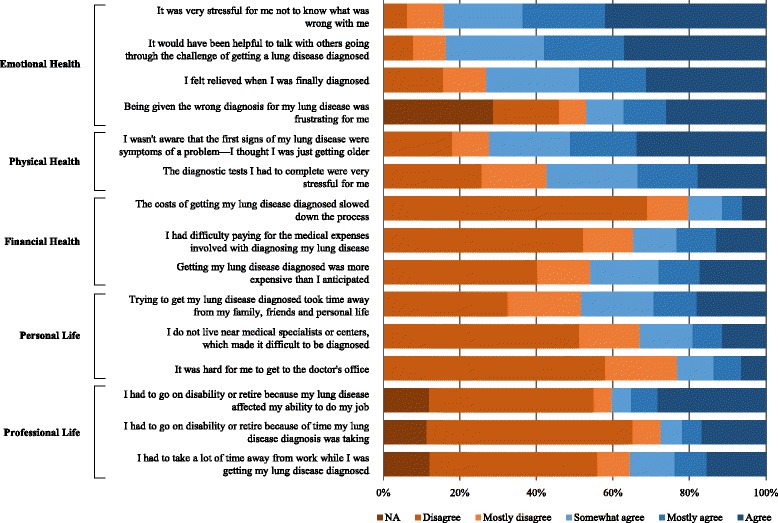
Quality of life assessments. Data are presented as the percentage of respondents for each response category

Most respondents indicated that the diagnostic process had an adverse impact on their quality of life (Fig. 6). More than 90% reported that the time required to obtain an accurate diagnosis had a meaningful effect on the amount of time spent with friends and family. Nearly half of all respondents reported difficulty finding time to attend doctor visits, with 28% reporting that time spent attending medical appointments and undergoing diagnostic procedures contributed at least in part to their decision to apply for disability benefits or retire. Diagnostic tests and procedures were commonly characterized as physically stressful, and more than 80% of respondents reported some degree of emotional stress due to the ongoing uncertainty regarding their diagnosis.

## Discussion

We have shown that the diagnostic experience for a substantial percentage of U.S. adults with ILD is characterized by repeated physician visits, multiple and often repeated diagnostic tests, frequent misdiagnosis, exposure to invasive procedures, and treatment with multiple therapies. Despite considerable progress in the understanding of IPF and other ILDs, these findings suggest a dichotomy in the diagnostic experience, with some patients receiving timely and accurate diagnoses, but a much larger proportion of patients experiencing significant challenges. This latter group the focus of the remaining discussion.

We identified several potential obstacles to a timely and accurate diagnosis. First, the common and non-specific nature of the symptoms often leads patients to dismiss initial symptoms and forego seeking medical attention. From the physician’s perspective, the typical presenting symptoms are more likely to be an indication of a common respiratory or cardiovascular condition, thus potentially leading to delays while such diagnoses are ruled out. Second, due to the diagnostic complexity of IPF and other ILDs, patients often undergo multiple diagnostic tests and procedures, including spirometry, serology, chest x-ray, HRCT, bronchoscopy, exercise tests, and surgical lung biopsy. Delays related to scheduling and availability, interpretation of test results, and the need to repeat certain tests and procedures contribute to the lengthy diagnostic process which, in turn, is reported to impose a considerable burden on patients and their caregivers. Indeed, a novel finding of the survey was the observation that 28% of respondents reported that the burden imposed by the diagnostic process was a factor in their decision to apply for disability benefits or retire. While further research is necessary to quantify the economic impact in terms of lost wages and reduced retirement benefits for both the patient and the primary caregiver, the observation that more than one-quarter of respondents reported that the diagnostic process was a contributing factor in the decision to retire or file for disability benefits suggests a need for meaningful improvements in diagnostic methods, timeliness, and accuracy. Finally, limited access or proximity to tertiary care-level expertise was commonly identified as an obstacle to obtaining a timely and accurate diagnosis. Notably, 68% of respondents cited referral to a subspecialist with expertise in ILD as the most important contributing factor to obtaining a clear diagnosis.

For patients with IPF who experience continued progression of disease during the diagnostic process, the consequences of a delay or misdiagnosis are particularly grave. Several studies have shown that reductions in forced vital capacity as small as 5–10% over 6 months are associated with a significant increase in the risk of death, [14–19] and delayed referral to subspecialty care has been shown to confer an increased risk of death in patients with IPF [20]. Additionally, a delay in obtaining an accurate diagnosis can delay evaluation for lung transplantation, potentially resulting in the loss of eligibility due to advanced age or frailty. Lastly, while the typical presenting symptoms of IPF mimic those of many other common pulmonary and cardiovascular disorders, they are uniquely unresponsive to agents frequently used in the treatment of other ILDs. Misdiagnosis therefore carries the added risk of exposure to ineffective or harmful therapies. In the present survey, one in five respondents with a current diagnosis of IPF reported prior treatment with a systemic corticosteroid—a potentially harmful therapy that carries a strong negative recommendation in the current international treatment guidelines [3, 21].

The findings of the survey are generally consistent with observations from two prior surveys of patients with pulmonary fibrosis [12, 22]. In a 2007 U.S. survey of patients with a physician-confirmed diagnosis of IPF, 55% of respondents reported a delay of at least 1 year between the initial onset of symptoms and a final diagnosis, and 38% reported consulting ≥ 3 physicians before a diagnosis of pulmonary fibrosis was established [22]. The most commonly reported misdiagnoses were bronchitis, asthma, and chronic obstructive pulmonary disease. A subsequent survey of IPF patients in five European countries reported similar findings [12]. Fifty-eight percent of patients experienced a delay of more than 1 year between initial presentation and a confirmed diagnosis of IPF, and more than half reported consulting ≥ 3 physicians before receiving a final diagnosis. The median time from initial presentation to confirmed diagnosis was 1.5 years. Asthma, chronic obstructive pulmonary disease, and pneumonia were the most commonly reported initial misdiagnoses. In the European survey, participants identified improved diagnostic techniques and improved access to tertiary care centers with expertise in ILD as the most common unmet needs.

In contrast to the previous surveys, more than 70% of respondents in the present survey were diagnosed after the 2011 publication of the current IPF diagnostic guidelines [3]. Comparison of results from the present survey with those from surveys conducted prior to the 2011 guidelines suggest that the new guidelines have had a marginal impact on the self-reported diagnostic experience of individuals with IPF. A lower proportion of respondents in the present survey experienced a ≥ 1-year delay in obtaining a clear diagnosis compared with the previous U.S. and European surveys (42% vs. 55% and 58%, respectively); however, a higher proportion of respondents in the present survey consulted ≥ 3 physicians compared with the previous surveys (75% vs. 38% and 55%, respectively). More than half of all respondents in both the current survey and the prior U.S. survey reported at least one misdiagnosis, with no evidence to suggest a change in the reported frequency of misdiagnosis following the revision of the guidelines. Additionally, while the current guidelines indicate that a pattern of usual interstitial pneumonia on HRCT is sufficient for a diagnosis of IPF under appropriate clinical circumstances, a surgical lung biopsy was performed in roughly half of all participants in both U.S. surveys, suggesting that clinical and radiological findings were inconclusive in as many as 50% of respondents. For those with severe disease or comorbidities, the increased risk might preclude surgery, resulting in an uncertain diagnosis and an inability to determine the optimal therapeutic intervention.

The results of the survey should be interpreted in the context of certain limitations. The data were collected from a non-random sample of adults living with ILD, the majority of whom were registered members of a national patient advocacy and support organization. The degree to which the findings may be generalized to a broader population is therefore unknown. Furthermore, prevalent rather than incident cases were sampled, which typically captures subjects with lesser disease severity and slower progression. Therefore, the survey might not have captured the most extreme cases of late diagnosis, as those diagnosed close to death were less likely to engage the Pulmonary Fibrosis Foundation or participate in the study. Additionally, subjects who had an unremarkable diagnostic experience or those in certain demographic groups might have also been less likely to participate in an online diagnostic survey, thereby introducing the potential for error due to non-response bias. Diagnoses and diagnostic procedures were self-reported by respondents and were not confirmed by medical records; accordingly, responses to these and other questions related to medical terms and procedures are subject to error due to misinformation bias. Finally, responses to questions requiring an accurate memory of historical details are subject to error due to recall bias. Despite these limitations, we believe the results of the survey provide important insights that will inform efforts to improve the diagnostic experience of patients with IPF and other ILDs.

## Conclusions

A nationwide survey of adults with ILD found that the diagnostic process is characterized by considerable delays, frequent misdiagnosis, exposure to costly and invasive diagnostic procedures, and substantial use of healthcare resources. Moreover, the experience is associated with meaningful adverse emotional, physical, social, and professional consequences for patients and their caregivers. These findings underscore the need for physician education, practical clinical guidelines, and improved diagnostic tools that increase the speed and accuracy of diagnosis and facilitate early therapeutic intervention.

## Additional files

Additional file 1: INTENSITY Survey Questions and Response Data. (DOCX 605 kb)

## Abbreviations

CT: Computed tomography
CTD: Connective tissue disease
FVC: Forced vital capacity
HRCT: High resolution computed tomography
ILD: Interstitial lung disease
IPF: Idiopathic pulmonary fibrosis
