# Supplementary material for: Barriers to timely diagnosis of interstitial lung disease in the real world: the INTENSITY survey
Source: BMC Pulm Med. 2018 Jan 17;18:9. doi: 10.1186/s12890-017-0560-x (PMC5773175; doi:10.1186/s12890-017-0560-x)
Supplement: Supplementary file 1 — INTENSITY Survey Questions and Response Data. (DOCX 605 kb) [file 12890_2017_560_MOESM1_ESM.docx]

**Barriers to Timely Diagnosis of Interstitial Lung Disease in the Real World: The Pulmonary Fibrosis Foundation INTENSITY Survey**

Gregory P. Cosgrove, MD, Pauline Bianchi, RN, Sherry Danese,

David J. Lederer, MD

Supplemental Material

INTENSITY Survey Questions

**INTRODUCTION**

*This research study is about your experiences and feelings throughout the time that your lung disease was being diagnosed. It will take approximately 20 minutes to complete. In appreciation for your time and effort, we are offering an honorarium of $20. Unfortunately, we cannot pay honoraria for surveys that are not fully completed***.**

**Question 1 Are you a U.S. resident?**

Yes

No

**Question 2: Which of the following types of lung disease has your doctor currently diagnosed you with?**

***Check all that apply***

Lung cancer

Asthma

Bronchitis or Chronic Bronchitis

Pneumonia

Another type of lung disease

I have not been diagnosed with a lung disease

I don't know

**Question 3 Are you currently diagnosed by your doctor with idiopathic pulmonary fibrosis (IPF)?**

Yes

No

**[ELIGIBLE SUBJECT INSTRUCTIONS]**

*Thank you, you qualify to participate in this survey.*

*The purpose of this survey is to understand your perspective on the diagnosis of your interstitial lung disease. Throughout this survey, we will refer to your lung disease as "interstitial lung disease." We will not be calling your specific type of interstitial lung disease by name, such as idiopathic pulmonary fibrosis (IPF), hypersensitivity pneumonitis (HP), connective tissue disease- associated interstitial lung disease (CTD-ILD), or sarcoidosis. When you read questions about "interstitial lung disease," in this survey, please think of the diagnosis your physician gave you.*

*If you wish to go back to a prior screen in the survey, please DO NOT use the back button on your browser. Instead, please use the arrow tabs at the bottom of the screen.*

**SURVEY QUESTIONS**

**Question 4 How long ago were you diagnosed with interstitial lung disease?**

***Please select one***

Within the last 6 months

7 months – 1 year

1–2 years

2–5 years

5–10 years

More than 10 years

**Question 5 What were the first symptoms of your interstitial lung disease?**

***Please check all that apply***

Shortness of breath or breathlessness

Cough

Fatigue and weakness

Discomfort in the chest

Unexplained weight loss

Loss of appetite

Other (please describe)

**Question 6 After you first noticed your symptoms, how long was it before you saw a doctor?**

***Please enter your answer in either months or years***

Months

Years

**Question 7 How many times did you see your primary care doctor before seeing a specialist about the symptoms you were experiencing?**

***Please check one***

1 time

2 times

3 times

4 times

>4 times

I did not see a primary care doctor before seeing a specialist

I did not see a specialist

I do not recall

**Question 8 Over the course of diagnosing your interstitial lung disease, which of the following types of doctors did you see?**

***Please check all that apply***

Family practice or general practice

Internal medicine

Pulmonologist (lung doctor)

Rheumatologist (doctor that treats arthritis and joints, muscles, and bones)

Cardiologist (heart doctor)

Thoracic surgeon (doctor who performs lung and chest biopsies and surgeries)

Other (please describe)

*Includes allergist (2%), gastroenterologist (1.7%), ENT (1.5%), infectious disease (1%), emergency medicine (8.3%)

**Question 9 Are any of these doctors in an Interstitial lung disease or pulmonary fibrosis center?**

***Please check one***

Yes

No

I don’t know

**Question 10** [If Q9=yes] **What is the name of the interstitial lung disease or pulmonary fibrosis center(s)?**

***Please enter name(s)***

**Question 11 In total, how many doctors did you see over the course of diagnosing your interstitial lung disease?**

***Please enter your answer***

**Question 12 In total, how much time did it take for you to receive your current diagnosis for interstitial lung disease? Please count from the time you first had symptoms of interstitial lung disease until the time you received your current diagnosis.**

***Please enter your answer in either months or years***

Months

Years

**Question 14 Over the course of diagnosing your interstitial lung disease, how much total time did you spend with an incorrect diagnosis?**

***Please enter your answer in either months or years***

Months

Years

**Question 15 Are you currently diagnosed with any of the following?**

**Please check all that apply**

Aging (no diagnosis)

Allergies

Asthma

Bronchiectasis

Bronchitis, bronchiolitis, or chronic bronchitis

Chronic obstructive pulmonary disease (COPD)

Cystic fibrosis

Emphysema

Gastroesophageal reflux disease (GERD)

Heart disease

Lung cancer

Need to exercise more

Obesity, overweight, or the need to lose weight

Pneumonia

Pulmonary edema

Pulmonary hypertension, pulmonary arterial hypertension, primary pulmonary hypertension

Sleep apnea

Tuberculosis

None of the above

**Comorbid conditions**

**Question 16 Over the course of diagnosing your interstitial lung disease, were you ever incorrectly diagnosed with any of the following?**

***Please check all that apply***

Aging (no diagnosis)

Allergies

Asthma

Bronchiectasis

Bronchitis, bronchiolitis, or chronic bronchitis

Chronic obstructive pulmonary disease (COPD)

Cystic fibrosis

Emphysema

Gastroesophageal reflux disease (GERD)

Heart disease

Lung cancer

Need to exercise more

Obesity, overweight, or the need to lose weight

Pneumonia

Pulmonary edema

Pulmonary hypertension, pulmonary arterial hypertension, primary pulmonary hypertension

Sleep apnea

Tuberculosis

None of the above

**Incorrect diagnoses**

**Question 17 What is your current diagnosis for interstitial lung disease?**

***Please check one***

Idiopathic pulmonary fibrosis (IPF)

Hypersensitivity pneumonitis (HP) or Chronic hypersensitivity pneumonitis

- Interstitial lung disease due to bird exposure
- Interstitial lung disease due to mold

Idiopathic non-specific interstitial pneumonia (NSIP) not due to a known cause

One of the following idiopathic interstitial pneumonias (IIP)

- Respiratory bronchiolitis–interstitial lung disease (RB-ILD)
- Desquamative interstitial pneumonia (DIP)
- Cryptogenic organizing pneumonia (COP)
- Lymphoid interstitial pneumonia (LIP)
- Idiopathic pleuroparenchymal fibroelastosis

Interstitial lung disease due to autoimmune or connective tissue disease (CTD-ILD)

- Rheumatoid arthritis associated interstitial lung disease (RA-ILD)
- Sjogrens disease associated interstitial lung disease
- Scleroderma or systemic sclerosis associated interstitial lung disease
- Lupus associated interstitial lung disease

Interstitial lung disease due to radiation therapy

Drug-related interstitial lung disease

Interstitial lung disease associated with an occupational exposure

- Asbestosis
- Coal worker's pneumoconiosis
- Silicosis

Sarcoidosis

One of the following interstitial lung diseases

- Vasculitis/diffuse alveolar hemorrhage (DAH)
- Langherhans cell histiocytosis (LCH)
- Lymphagioleiomyomatosis (LAM)
- Pulmonary alveolar proteinosis (PAP)
- Eosinophilic pneumonias
- Neurofibromatosis
- Familial interstitial pneumonia
- Chronic aspiration

A different type of interstitial lung disease (please describe)

*Includes vasculitis/diffuse alveolar hemorrhage, Langherhans cell histiocytosis, lymphagioleiomyomatosis, pulmonary alveolar proteinosis, eosinophilic pneumonias, neurofibromatosis, familial interstitial pneumonia, and chronic aspiration.

**Question 18 Over the course of diagnosing your interstitial lung disease, were you ever incorrectly diagnosed with any of the following?**

***Please check all that apply***

Idiopathic pulmonary fibrosis (IPF)

Hypersensitivity pneumonitis (HP) or Chronic hypersensitivity pneumonitis

- Interstitial lung disease due to bird exposure
- Interstitial lung disease due to mold

Idiopathic non-specific interstitial pneumonia (NSIP) not due to a known cause

One of the following idiopathic interstitial pneumonias (IIP)

- Respiratory bronchiolitis–interstitial lung disease (RB-ILD)
- Desquamative interstitial pneumonia (DIP)
- Cryptogenic organizing pneumonia (COP)
- Lymphoid interstitial pneumonia (LIP)
- Idiopathic pleuroparenchymal fibroelastosis

Interstitial lung disease due to autoimmune or connective tissue disease (CTD-ILD)

- Rheumatoid arthritis associated interstitial lung disease (RA-ILD)
- Sjogrens disease associated interstitial lung disease
- Scleroderma or systemic sclerosis associated interstitial lung disease
- Lupus associated interstitial lung disease

Interstitial lung disease due to radiation therapy

Drug-related interstitial lung disease

Interstitial lung disease associated with an occupational exposure

- Asbestosis
- Coal worker's pneumoconiosis
- Silicosis

Sarcoidosis

One of the following interstitial lung diseases

- Vasculitis/diffuse alveolar hemorrhage (DAH)
- Langherhans cell histiocytosis (LCH)
- Lymphagioleiomyomatosis (LAM)
- Pulmonary alveolar proteinosis (PAP)
- Eosinophilic pneumonias
- Neurofibromatosis
- Familial interstitial pneumonia
- Chronic aspiration

A different type of interstitial lung disease (please describe)

I have never been misdiagnosed with another interstitial lung disease

*Includes vasculitis/diffuse alveolar hemorrhage, Langherhans cell histiocytosis, lymphagioleiomyomatosis, pulmonary alveolar proteinosis, eosinophilic pneumonias, neurofibromatosis, familial interstitial pneumonia, and chronic aspiration.

**Question 19 What type of doctor provided you with your current diagnosis for interstitial lung disease?**

***Please check one***

Family practice or general practice

Internal medicine

Pulmonologist (lung doctor)

Rheumatologist (doctor that treats arthritis and joints, muscles, and bones)

Cardiologist (heart doctor)

Thoracic surgeon (doctor who performs lung, chest, and heart surgeries and biopsies)

Other (please describe)

**Question 20 Is this doctor in an Interstitial Lung Disease (ILD) or Pulmonary Fibrosis (PF) Center?**

***Please check one***

Yes

No

I don’t know

**Question 21** [If Q20=yes] **What is the name of the Interstitial Lung Disease (ILD) or Pulmonary Fibrosis (PF) Center?**

***Please enter name***

**Question 22 Do you feel that your current diagnosis is a clear and final diagnosis?**

***Please check one***

Yes

No

**Question 23 What type of doctor treats your interstitial lung disease today? If you see more than one doctor for interstitial lung disease, please select the type of doctor who you feel is the decision-maker about your treatment and care.**

***Please check one***

Family practice or general practice

Internal medicine

Pulmonologist (lung doctor)

Rheumatologist (doctor that treats arthritis and joints, muscles, and bones)

Cardiologist (heart doctor)

Thoracic surgeon (doctor who performs lung, chest, and heart surgeries and biopsies)

Other (please describe)

**Question 24 Is this doctor in an Interstitial Lung Disease (ILD) or Pulmonary Fibrosis (PF) Center?**

***Please check one***

Yes

No

I don’t know

**Question 25** [If Q20=yes] **What is the name of the Interstitial Lung Disease (ILD) or Pulmonary Fibrosis (PF) Center?**

***Please enter name***

**OBSTACLES AND CHALLENGES**

**Question 26 Please rate how strongly you agree with each of the following statements about your experience being diagnosed with interstitial lung disease.**

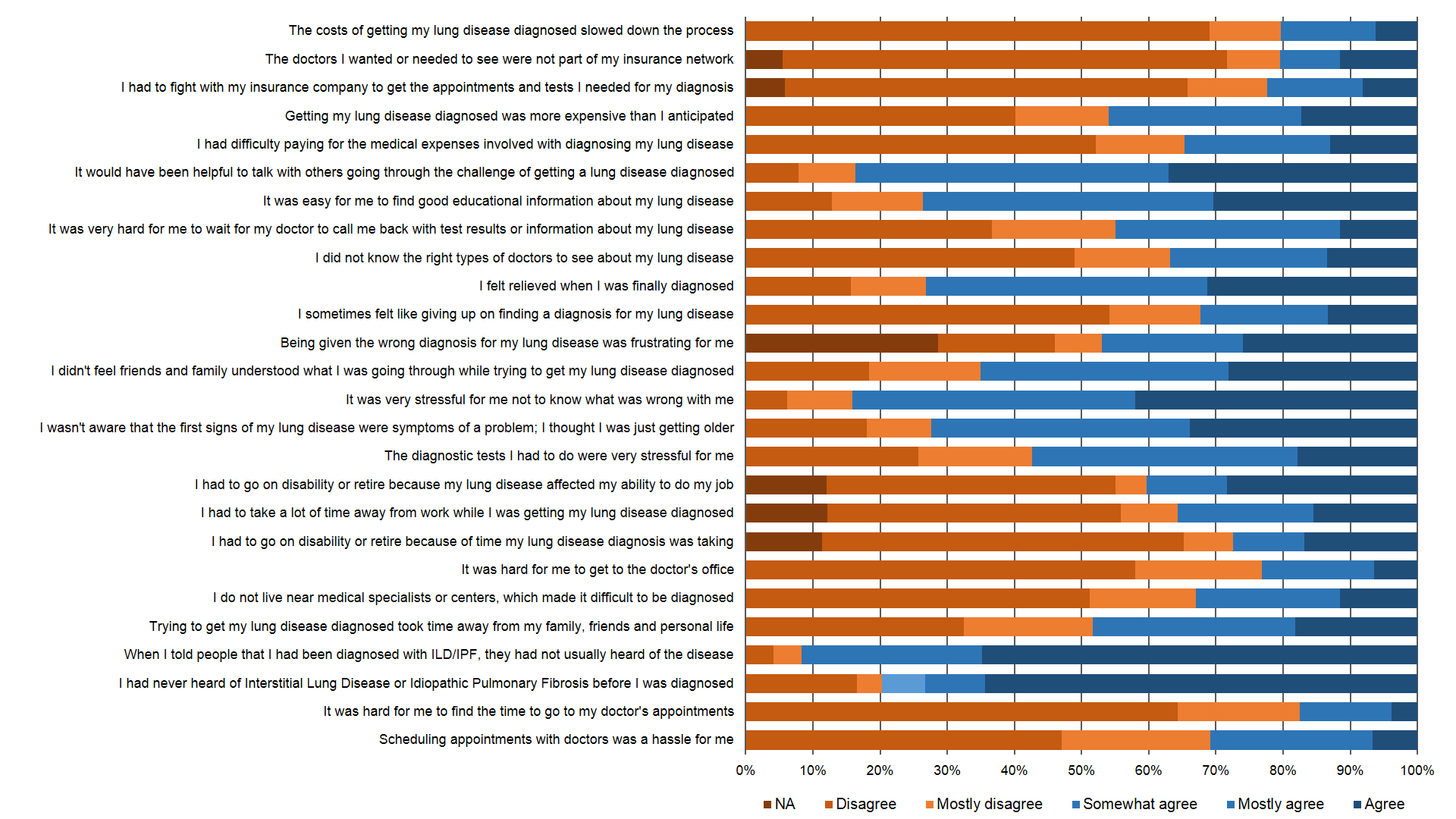


**Question 27 Please think back to all of the different diagnostic tests you had while your interstitial lung disease was being diagnosed. Which of the following did you have?**

***Please check all that apply***

Bronchoscopy (a tube is placed into your mouth or nose and threaded into your lungs)

Chest x-ray

Computed tomography (CT scan)

High resolution computed tomography (HRCT scan)

Echocardiogram (uses sound waves to look at the heart)

Surgical lung biopsy (a surgeon takes a sample of lung tissue by making an incision in your chest while you are under general anesthesia)

Oximetry (a small device is placed on a finger to measure oxygen saturation in your blood)

Pulmonary function testing (you blow as hard and as long as possible through a tube connected to a machine that measures the air moving in and out of your lungs)

Six-minute walk test (measures how far you can walk in 6 minutes)

**Question 28 How many times have you had each of those tests in the course of diagnosing your interstitial lung disease?**

***Please indicate the number of times for each test***

Bronchoscopy (a tube is placed into your mouth or nose and threaded into your lungs)

Chest x-ray

Computed tomography (CT scan)

High resolution computed tomography (HRCT scan)

Echocardiogram (uses sound waves to look at the heart)

Surgical lung biopsy (a surgeon takes a sample of lung tissue by making an incision in your chest while you are under general anesthesia)

Oximetry (a small device is placed on a finger to measure oxygen saturation in your blood)

Pulmonary function testing (you blow as hard and as long as possible through a tube connected to a machine that measures the air moving in and out of your lungs)

Six-minute walk test (measures how far you can walk in 6 minutes)

**Question 29 Which of the following medications or treatments are you currently taking?**

***Please check all that apply***

Aciphex (also called rabeprazole)

Advair (also called salmeterol plus fluticasone)

Aerobid, Aerospan HFA, Aerobid-M (also called flunisolide)

Alupent or Metaprel (metaproterenol)

Antibiotics

Atrovent (also called ipratropium bromide)

Cellcept (also called mycophenolate mofetil)

Cytoxan (also called cyclophosphamide)

Dexilant or Kapidex (also called dexlansoprazole)

Dulera inhaler (also called formoterol plus mometasone)

EpiPen, Adrenalin, EpiPen 2-Pak, Auvi-Q (also called epinephrine)

Esbriet (also called pirfenidone)

Flovent, Flovent HFA, Arnuity Ellipta, Flovent Diskus (also called fluticasone)

Imuran (also called azathioprine)

Lifestyle changes (quit smoking, healthy diet, plenty of rest, practice relaxation techniques)

Medrol Dosepak, Solu-Medrol, Medrol, MethylPREDNISolone Dose Pack (also called methylprednisone)

N-acetyl cysteine (also called N-acetyl-L-cysteine or NAC)

Nexium (also called esomeprazole)

Ofev (also called nintedanib)

Orapred, Prelone, PediaPred, Predicort RP (also called prednisalone)

Prednisone, Deltasone, Rayos, Sterapred, Meticorten

Prevacid (also called lansoprazole)

Prilosec, Zegerid, Omesec (also called omeprazole)

Protonix (also called pantoprazole)

Pulmonary rehabilitation

Symbicort (also called formoterol plus budesonide)

Uceris, Pulmicort Turbohaler, Entocort EC, Pulmicort Flexhaler (also called budesonide)

Vanceril, Beclovent or Qvar (also called beclomethasone)

Ventolin, Proventil, or ProAir (also called albuterol)

I am in a clinical trial

I am in a lung transplant program

I am not currently taking any medications or treatments

Other medications or treatments for your interstitial lung disease (please describe)

*See next page for response summary*

**Current Medications/Treatments**

**Question 30 Which of the following medications have you taken in the past, but are no longer taking?**

***Please check all that apply***

Aciphex (also called rabeprazole)

Advair (also called salmeterol plus fluticasone)

Aerobid, Aerospan HFA, Aerobid-M (also called flunisolide)

Alupent or Metaprel (metaproterenol)

Antibiotics

Atrovent (also called ipratropium bromide)

Cellcept (also called mycophenolate mofetil)

Cytoxan (also called cyclophosphamide)

Dexilant or Kapidex (also called dexlansoprazole)

Dulera inhaler (also called formoterol plus mometasone)

EpiPen, Adrenalin, EpiPen 2-Pak, Auvi-Q (also called epinephrine)

Esbriet (also called pirfenidone)

Flovent, Flovent HFA, Arnuity Ellipta, Flovent Diskus (also called fluticasone)

Imuran (also called azathioprine)

Lifestyle changes (quit smoking, healthy diet, plenty of rest, practice relaxation techniques)

Medrol Dosepak, Solu-Medrol, Medrol, MethylPREDNISolone Dose Pack (also called methylprednisone)

N-acetyl cysteine (also called N-acetyl-L-cysteine or NAC)

Nexium (also called esomeprazole)

Ofev (also called nintedanib)

Orapred, Prelone, PediaPred, Predicort RP (also called prednisalone)

Prednisone, Deltasone, Rayos, Sterapred, Meticorten

Prevacid (also called lansoprazole)

Prilosec, Zegerid, Omesec (also called omeprazole)

Protonix (also called pantoprazole)

Pulmonary rehabilitation

Symbicort (also called formoterol plus budesonide)

Uceris, Pulmicort Turbohaler, Entocort EC, Pulmicort Flexhaler (also called budesonide)

Vanceril, Beclovent or Qvar (also called beclomethasone)

Ventolin, Proventil, or ProAir (also called albuterol)

I have not taken any of these medications

*See next page for response summary*

**Previous Medications/Treatments**

**Question 31 Where did you find the most useful information about interstitial lung disease?**

***Please check up to 3***

Your doctor

Nurses or physicians' assistants in your doctor's office

Interstitial Lung Disease (ILD) or Pulmonary Fibrosis (PF) Center

In the hospital

Friends and family members

Support groups

Pulmonary Fibrosis Foundation

Care Center Network

Mayo Clinic website

WebMD website

Wikipedia website

Cleveland Clinic website

Medicine Net website

Right Diagnosis website

National Jewish Health website

The American Lung Association website

Medscape website

eMedicine website

Dr. Jeff Swigris blog

Dr. Dave Lederer blog

Other websites

Newspapers (print or websites)

I don't know

Other (please describe)

**Question 32 What do you think were the most important things that helped you to get interstitial lung disease diagnosed clearly?**

***Please check up to 3***

Seeing a doctor that specialized in your type of lung disease

Working with a nurse or physicians' assistant in your doctor's office

Being seen at an Interstitial Lung Disease or Idiopathic Pulmonary Fibrosis Center

Getting the high resolution computed tomography (HRCT scan)

Having an expert radiologist who specializes in this type of lung disease look at HRCT scan

Getting pulmonary lung function testing

Getting the bronchoscopy

Getting the surgical lung biopsy

My own research about lung disease

I do not feel that I have a clear diagnosis yet

**Question 33 Were you exposed to any of the following before you began to experience interstitial lung disease symptoms?**

***Please check all that apply***

Aerospace industry

Asbestos

Azulfidine, Diamox, Gantrisin, Sulfazine, Truxazole, or Zonegran (also called sulfonamide)

Chemotherapy

Coal dust

Cordarone, Pacerone, Cordarone IV, or Nexterone (also called amiodarone)

Dilantin, Phenytoin Sodium, Phenytek (also called phenytoin)

Excavation

Farming or farm dust

Foundry work

Fumes from welding, smelting, furnace work, rubber manufacturing or pottery making

Heavy metals in the workplace

Inderal, Inderal LA, InnoPran XL, or Hemangeol (also called propranolol)

Indoor hot tubs

MacroBid (also called nitrofurantoin)

Mining

Mold in your home or workplace (other than the bathroom)

Pet birds within 10 years prior to the onset of symptoms or diagnosis

Quarry work

Radiation therapy

Silica dust

Stone work

I don't know

None of the above

**Exposure History**

**Question 34 Does anyone in your family have interstitial lung disease?**

***Please check one***

Yes

No

**Question 35 What is your gender?**

***Please check one***

Male

Female

**Question 35 What is your age?**

***Please type in your answer***

**Age, years**

**Question 37 What is your current employment status?**

***Please check one***

Full-time work

Part-time work

Retired

On disability

**Question 38 What is your household annual income?**

***Please select one***

$0 - $25,000

$25,000 - $50,000

$50,000 - $75,000

$75,000 - $100,000

$100,000 or more

Prefer not to reply
